# Telehealth and the work behavior of mental health clinicians

**DOI:** 10.1093/haschl/qxag070

**Published:** 2026-03-25

**Authors:** Sooyeon Song, Steven D Pizer, Christine A Yee

**Affiliations:** Department of Health Law, Policy, and Management, Boston University School of Public Health, Boston, MA 02118, United States; Partnered Evidence-based Policy Resource Center, Boston VA Healthcare System, Boston, MA 02130, United States; Department of Health Law, Policy, and Management, Boston University School of Public Health, Boston, MA 02118, United States; Partnered Evidence-based Policy Resource Center, Boston VA Healthcare System, Boston, MA 02130, United States; Department of Health Law, Policy, and Management, Boston University School of Public Health, Boston, MA 02118, United States; Partnered Evidence-based Policy Resource Center, Boston VA Healthcare System, Boston, MA 02130, United States

**Keywords:** telehealth, clinician behavior, Veterans health

## Abstract

**Introduction:**

Telehealth delivers more than half of mental health care in the United States amid persistent mental health workforce shortages. However, little is known about how telehealth relates to clinicians’ work behavior, specifically their visits per clinic day and probability of turnover.

**Methods:**

We examined mental health clinicians working at the Veterans Health Administration who collectively delivered 26 million visits. We regressed clinicians’ monthly visit volume (supply), number of clinic days worked (input), visits per clinic day (throughput), and probability of turnover (retention) on the proportion of their mental health visits delivered via video. We used variation in broadband availability as an instrumental variable for video visit use.

**Results:**

A 10 percentage-point higher proportion of video visits was associated with no significant difference in total visits, 0.9 fewer clinic days per month (8.5% of the sample mean), 0.5 more visits per clinic day (6.2% of the sample mean), and a 0.4 percentage-point lower probability of monthly turnover (46% of the sample mean). Among psychologists and social workers, higher video share was associated with more visits per clinic day and fewer clinic days, whereas psychiatrists showed fewer visits per clinic day.

**Conclusion:**

Telehealth may shape clinicians’ work behavior, including visits per clinic day, allocation of clinic days, and retention.

## Introduction

The health care workforce has declined substantially over the past 5 years, with particularly severe losses in mental health services.^1,2^ In 2024, more than one-third of the US population lived in areas with too few mental health professionals.^3^ Nearly half of adults with a mental illness did not receive treatment in 2023, a gap that stems partly from limited supply.^4^

Telehealth may help mitigate these supply constraints.^5-8^ During the COVID-19 pandemic, it accounted for 60%-90% of mental health visits, and 13% of clinicians practiced exclusively via telehealth.^9-11^ Prior studies report that more mental health visits occur in practices with greater telehealth use.^12,13^ However, evidence on how telehealth relates to clinician supply dynamics remains limited.

This uncertainty persists because telehealth could affect clinician workflow through multiple channels that may offset one another. Studies have shown that telehealth is associated with longer electronic health record documentation times,^14^ fewer no-show appointments,^15^ and shorter travel time for clinicians,^16^ but research has not yet clarified whether telehealth changes the number of visits a clinician provides per day or the number of days that the clinician works. One study using 2018 data showed higher productivity, measured as relative value units per full-time equivalent, following telehealth adoption.^17^ Telehealth has also been associated with reduced self-reported burnout,^18^ yet there is no clear evidence on turnover. As telehealth continues to account for up to half of mental health care, and recent federal regulations have again extended temporarily flexibilities for telehealth use,^19^ understanding its relationship with clinician supply has implications for telehealth policy and workforce planning.

To address this need, our objective was to examine differences in 4 supply characteristics by telehealth share: total visits delivered per month, number of clinic days per month, number of visits per clinic day, and turnover. We analyzed mental health clinicians employed by the Department of Veterans Affairs (VA), where salaried compensation limits the influence of reimbursement incentives on telehealth use. Because clinicians’ unobserved characteristics, such as preferences for care modality, may correlate with both telehealth use and supply outcomes, we applied a quasi-experimental, instrumental variable (IV) approach to estimate differences in clinicians’ supply by the extent of telehealth provision.

## Methods

### Data

We primarily used administrative data from the VA Corporate Data Warehouse covering outpatient mental health encounters between January 1, 2019, and December 31, 2021. These data contains encounter-level information including service dates, stop codes (VA 3-digit codes that define place of service and procedure category), and the clinician who provided the encounter. We supplemented the data with (1) Federal Communications Commission (FCC) county-level data on broadband availability, including types of broadband technology and available download speeds; (2) VA payroll records on clinician characteristics, including graduation year and biweekly gross pay; (3) Area Health Resource Files, which provide annual county-level health care market characteristics, including population size and physician density; and (4) Centers for Disease Control and Prevention data on monthly county counts of COVID-19 cases. All variables were merged at the most granular unit available of observation. This evaluation was conducted as a quality improvement activity for VHA and was deemed by the Research & Development committee at the VA Boston Healthcare System not to be research; therefore, it was not subject to Institutional Review Board review.

### Sample

The sample included psychologists, social workers, and psychiatrists/neurologists (combined in VA clinician classification) who provided mental health visits to a defined patient cohort between 2019 and 2021. These patients were those who had an outpatient mental health visit (defined by VA stop codes^9^) in 2019, which we refer to as the *patient cohort*. Using a patient cohort defined before COVID-19 helped control for changes in patient composition due to COVID-19. The 3 clinician categories accounted for more than 70% of all mental health visits, totaling approximately 26 million encounters. We excluded clinician-months with fewer than 5 visits to reduce noise from residual administrative or miscoded encounters. Trainees (interns, residents, and fellows) and clinicians with fewer than 5 years since graduation were also excluded to avoid instability in supply outcomes from early career practice patterns.

### Variables

#### Exposure

For each clinician, we measured the proportion of mental health visits delivered to the patient cohort via video telehealth. The denominator included visits that were synchronous patient-clinician contacts by video, phone, or in-person visits. We focused on video because it provides visual information beyond audio-only visits and is more comparable to in-person care.^19^ The proportion of video visits was lagged by 3 months to account for temporal delays in supply outcomes.

#### Outcomes

We evaluated 4 measures: (1) total number of visits that a clinician provided in a month, defined as the sum of all outpatient mental health encounters (in-person, phone, video) to the patient cohort; (2) number of 8-hour clinical practice days a clinician provided in a month, calculated by summing the hours between the first and last mental health visit each day in a given month and dividing by 8; (3) monthly average number of visits per clinic day, calculated by dividing the total number of visits by the number of clinic days; and (4) whether a clinician departed from active outpatient mental health practice in the VA in a given month (ie, turnover), a binary indicator equal to 1 if the clinician was not observed to provide mental health visits in the subsequent 12 months. The clinic days measure reflects clinical availability for patient care. It does not distinguish between direct encounters, documentation, administrative tasks, or gaps between visits. The turnover measure may reflect leaving VA employment, transitioning to nonclinical roles, or taking an extended leave of absence.

#### Covariates

We included clinician-, facility-, and market-level covariates that might be related to clinician supply outcomes. Clinician covariates were a female indicator, years since graduation (tenure), and hourly wage. Hourly wage was calculated as biweekly gross pay divided by paid hours, winsorized at the top and bottom 1% of the clinician-month distribution to address potential outliers, and then adjusted to 2020 dollars. Our facility covariate was the turnover rate of registered nurses (RNs), calculated as the number of nurses who met our turnover definition above in a facility in each month divided by the number of nurses practicing in that facility. This measure controls for the overall work environment. Local market covariates were population (in 100 000 s) (local demand), per capita income (affluence), physicians per 100 000 residents (local supply, including VA and non-VA physicians), the unemployment rate (socioeconomic conditions), and COVID-19 case counts per 100 000 residents. The market-level covariates were derived from county-level data, which we aggregated to the market (or medical center) level using enrollee-weighted averages. All models included region fixed effects (Veterans Integrated Service Networks [VISN]), year fixed effects, and month fixed effects. The VA VISN regions determine the allocation of the national VA budget, and policies affecting clinician workload are set by VISN directors and implemented at the VISN level. Year fixed effects control for nationwide differences in video use and clinician work behavior across years, while month fixed effects control for nationwide seasonal patterns.

#### Instrumental variable

We used broadband availability as an instrument for the proportion of video visits. Patients with broadband access are more likely to use mental health video visits.^20,21^ Broadband availability was measured as the proportion of residents in a patient's county of residence with access to fiber-optic internet at certain download speeds (0-1 scale, where 1 indicates that 100% of the residents have access). We aggregated this measure to the clinician-month level by averaging broadband availability across patient visits to that clinician during the month (see eMethod for details). The resulting variation in the instrument reflects patient broadband availability and is assumed to be exogenous to the clinician's unobserved supply characteristics. Fiber-optic internet was selected because fiber requires greater infrastructure investment than other technologies (eg, satellite and cellular), exhibits greater cross-county variation, and is less susceptible to short-term policy shocks such as COVID-19-related broadband expansions. Following FCC guidance that 25 megabits per second (Mbps) is the minimum recommended download speed for video conferencing, we constructed instruments for 25, 100, and 250 Mbps. We selected 250 Mbps as the preferred instrument given its sufficient cross-clinician and over-time variation for estimation (Figures S1-S3). Results were similar when using 25 and 100 Mbps (Table S1).

The instrument improves balance in clinician-level covariates and some of the market-level covariates, supporting the use of the instrument (Table S2, Panel B). Table S2 shows the covariate differences between clinicians with above-average video visit rates and those with below-average video rates (Panel A), and the covariate differences between clinicians with patients who have above-average broadband and those who have below-average broadband (Panel B). Compared to the stratification by observed video provision, stratification by broadband availability reduced standardized differences in 4 of the 9 covariates. Among these, 5 fell below the 0.10 threshold, while COVID-19 burden decreased but remained above 0.10. Four covariates remained above 0.10.

In addition to controlling for these covariates in our models, we designed the instrument to minimize the likelihood of correlation with unobserved confounders conditional on observed covariates. First, broadband availability was defined at patients’ counties of residence, which is less likely to be associated with clinician-level unobservables than measures based on practice location. Second, using broadband availability rather than uptake reduces correlation with county affluence, as purchasing behavior may reflect local affluence. Broadband availability reflects supply-side infrastructure deployment in the data generating process and is therefore less directly linked to household purchasing behavior. Third, instrument variation reflects both residential mobility (approximately 15% of patients moved counties) and changes in broadband within fixed residences, making it unlikely to be correlated with clinician supply characteristics.

We examined whether broadband availability is associated with overall visit volume or only visit composition, as changes in overall volume would suggest a violation of the exclusion restriction. Broadband availability was positively correlated with the number of video visits and negatively correlated with the number of phone and in-person visits, with the combined decreases being similar in magnitude to the video increase (Table S3). This pattern suggests substitution across visit modalities rather than a change in overall visit volume, consistent with prior studies.^9,22^

Our identification strategy relies on within-region variation in the instrument using a pooled cross-section of clinician-month observations. All models control for time-varying market and facility characteristics, including population, per capita income, physician supply, unemployment, and nurse turnover rate. With region fixed effects and other covariates, the first-stage *F*-statistic is 539.9, rejecting the null of a weak instrument. With more granular fixed effects, we cannot reject the null of a weak instrument: the first-stage *F*-statistic is 1.1 with facility fixed effects and 3.0 with clinician fixed effects (Table S4, Specifications 5 and 6), indicating that within-facility variation is insufficient for identification.

### Statistical analysis

We examined how changes in the proportion of video visits relate to clinician outcomes using two-stage least-squares (2SLS). In the first stage, broadband availability predicted the proportion of video visits; the predicted proportion of video visits from this stage was used to estimate the change in outcomes in the second stage. We also used the Hausman test to compare the 2SLS estimates to ordinary least squares (OLS) estimates. We additionally ran the 2SLS regressions separately for each of the 3 clinician categories to examine heterogeneous effects. All models adjust for covariates described above. All *P*-values were from two-sided tests, and results were considered statistically significant at *P* < 0.05.

We tested the robustness of the results in the following cohorts and specifications: (1) clinicians who provided at least 1 mental health visit during 2019-2021, capturing variation from new entrants; (2) patients with at least 1 mental health visit during 2019-2021, an open patient cohort that captures changes in patient characteristics during the pandemic and expanded telehealth access; (3) all clinicians with at least 1 visit during the study period, relaxing our preferred threshold of 5 visits and thereby including low-volume clinicians or clinician-months with potential data anomalies; and (4) models using concurrent rather than 3-month lagged video share, to assess whether results are sentive to the assumed temporal delay.

Our 2SLS estimates are identified from the portion of clinicians’ video use explained by their patients’ broadband availability. For some clinicians, video visit rates are predicted by their patients’ broadband availability, whereas for others they may be driven primarily by clinician preferences. To characterize the former group, we applied a kappa-weighting approach.^23,24^ We dichotomized both video visit share and broadband availability at their sample medians to construct binary treatment and instrument indicators and calculated clinician-level weights. The largest weights were assigned to 2 groups: (1) clinicians with video visit shares above the median and patient broadband above the median, and (2) clinicians with video visit shares below the median and patient broadband below the median. Clinicians outside these groups received smaller weights determined by the predicted probability of above-median broadband based on the covariates. The first-stage *F*-statistic of this dichotomized model exceeds the Stock-Yogo critical value for weak instruments. Standard errors were obtained using 1000 bootstrap replications.

### Limitations

First, our identification relies on variation across clinicians within region, year, and month. The instrument becomes weak in the first stage when facility or clinician fixed effects are included. Although we control for local area characteristics such as income and population, and although the instrument improves balance in clinician-level covariates, unobserved area-level factors within regions may still confound the estimates. Second, our models did not explicitly adjust for patient risk, visit intensity, or quality. Although we examined outpatient mental health visits within a closed patient cohort, which reduces variation in patient mix and visit intensity, results should be interpreted with these limitations in mind. Third, we restricted the study period to 2019-2021 because FCC data adopted new reporting formats after 2021, limiting longitudinal comparability.

## Results

The sample included 226 153 clinician-months (10 814 unique clinicians). Clinicians above the median proportion of video visits were more often female, had shorter tenure, and earned lower hourly wages compared with those below the median, and they were more likely to practice in areas with greater physician supply and higher COVID-19 burden (Panel A of Table 1). By clinician type, 87 605 (38.7% of the sample, 4025 unique psychologists) of clinician-months were psychologists (mean 16 years of tenure, 65% female, mean hourly wage $86), 75 683 (33.5%, 3951 unique social workers) were social workers (17.3 years, 75% female, $62), and 62 865 (27.8%, 2838 unique psychiatrists/neurologists) were psychiatrists/neurologists (24 years, 48% female, $180) (Panel B of Table 1). Visits per clinic day declined for psychologists and social workers at the onset of the pandemic and had not fully recovered by 2021, whereas psychiatrist/neurologist visits per clinic day were relatively stable, though gradually decreasing throughout the sample period. Turnover spiked at the onset of the pandemic across all 3 clinician types (Figure S4).

**Table 1.** Clinician characteristics by median video visit proportion and clinician type.

		A. Clinician characteristics stratified by median proportion of video visits					B. Clinician characteristics stratified by clinician type					
		Below median		Above median (greater video visit)			Psychologist		Social worker		Psychiatrist	
		(*N* = 113 101)		(*N* = 113 052)			(*N* = 87 605)		(*N* = 75 683)		(*N* = 62 865)	
		Mean	SD	Mean	SD	*P* value	Mean	SD	Mean	SD	Mean	SD
Clinician	Tenure years since graduation (year)	18.90	(10.72)	17.99	(9.55)	<0.001	15.63	(8.49)	17.29	(8.81)	23.75	(11.68)
	Female (0-1)	0.61	(0.49)	0.66	(0.47)	<0.001	0.65	(0.48)	0.75	(0.43)	0.48	(0.50)
	Hourly wage (dollar)	105.31	(63.21)	103.33	(58.04)	<0.001	86.36	(35.00)	61.93	(21.07)	180.38	(51.08)
Facility	RN turnover rate (0-1)	0.04	(0.09)	0.04	(0.09)	0.004	0.04	(0.09)	0.04	(0.09)	0.04	(0.09)
Market	Population (in 100 000 s)	6.55	(7.63)	6.17	(7.34)	<0.001	6.58	(7.38)	5.51	(7.08)	7.08	(8.00)
	Per capita income (dollar)	52 816	(13 214)	56 074	(13 326)	<0.001	55 287	(13 596)	53 480	(12 264)	54 432	(14 220)
	Physicians (in 100 000 s)	0.17	(0.23)	0.17	(0.22)	<0.001	0.17	(0.22)	0.14	(0.19)	0.20	(0.27)
	Unemployment rate (%)	4.63	(2.04)	6.16	(2.18)	0.01	5.39	(2.24)	5.30	(2.19)	5.53	(2.31)
	COVID-19 cases (in 100 000 s)	0.03	(0.11)	0.17	(0.26)	<0.001	0.10	(0.21)	0.09	(0.19)	0.11	(0.23)

Panel A compares clinician, facility, and market characteristics by median video visit proportion. Panel B compares characteristics across 3 clinician types.

Our first-stage results show that the instrument is correlated with the proportion of video visits. Clinicians whose patients resided in counties with higher broadband availability showed a higher share of video visits (Table S3). The Kleibergen–Paap Wald rk *F*-statistic was 539.9, exceeding the Stock and Yogo critical value (16.38) for 1 endogenous variable, 1 IV, and less than 5% maximal IV size distortion relative to the 5%-level Wald test, indicating that the instrument is strong.^25^

In the overall sample, the 2SLS estimates show that higher video visit rates do not change total visits in a statistically significant way. Higher video visit rates, however, are associated with fewer clinic days, more visits per clinic day, and a lower likelihood of turnover (Table 2, Panel B). Specifically, a 10 percentage-point higher video visit share was associated with 0.89 fewer clinic days per month (95% confidence interval [CI], −1.07 to −0.70; *P* < 0.001), 0.54 more visits per clinic day (95% CI, 0.37-0.71; *P* < 0.001), and a 0.4 percentage-point lower probability of turnover (95% CI, −0.7 to −0.1; *P* < 0.05).

**Table 2.** Clinician work behavior measures by clinician video visit rates.

	A. Multivariable regression		B. Instrumental variable regression		C. *P* value
	Coeff.	95% CI	Coeff.	95% CI	
**Number of total visits**					
Coefficient	−9.18***	(−9.96, −8.41)	−8.04	(−24.22, 8.14)	0.89
Elasticity	−0.03		−0.02		
**Clinic days**					
Coefficient	0.16***	(0.07, 0.25)	−8.86***	(−10.73, −6.98)	<0.001
Elasticity	0.00		−0.19		
**Visits per clinic day**					
Coefficient	−1.17***	(−1.25, −1.09)	5.36***	(3.68, 7.05)	<0.001
Elasticity	−0.03		0.14		
**Probability of turnover**					
Coefficient	0.00	(−0.00, 0.00)	−0.04*	(−0.07, −0.01)	0.01
Elasticity	0.04		−1.04		
All covariates	X		X		
Region fixed effects	X		X		
Year fixed effects	X		X		
Month fixed effects	X		X		
First-stage *F*-statistics	—		539.9		

^***^ *P* < 0.001, ** *P* < 0.01, * *P* < 0.05. Each coefficient represents the estimated change in the outcome associated with moving from 0 to 1 in the share of video visits (a complete shift from in-person to video visits). To rescale to a 10% point increase in video visit share, divide the coefficient by 10 (eg, 5.36/10 = 0.54 more visits per clinic day). The elasticities are the percentage change in the outcome (relative to its sample mean) for a 1% change in the sample mean of the video visit share. All models include region fixed effects, year fixed effects, month fixed effects, and the full covariate set. Covariates include clinician characteristics (female, tenure, hourly wage), facility characteristics (RN turnover rate), and market characteristics (population, per capita income, unemployment rate (%), physicians, COVID-19 cases). Column C reports *P* values from a Durbin–Wu–Hausman specification test comparing the multivariable and instrumental variable estimates.

Compared to OLS estimates, the 2SLS estimates were larger in magnitude and sometimes reversed the sign of the estimate. For clinic days, OLS showed a positive association while 2SLS showed a negative estimate; for visits per clinic day, OLS showed a negative association while 2SLS showed a positive estimate. Covariate estimates were consistent with expectations in sign and significance (Table S5). The Hausman test rejected the null hypothesis of consistency of the OLS estimates for clinic days (*P* < 0.001), visits per clinic day (*P* < 0.001), and turnover (*P* = 0.01), but not for total visits (*P* = 0.89) (Panel C of Table 2).

The OLS estimates reflect the overall correlation between video visit rates and clinician supply measures after controlling for clinician, facility, and market characteristics. This OLS estimate equally weights all clinicians and does not address potential selection into video use. The 2SLS estimates instead identify variation in video use explained by patients’ broadband availability. This approach reduces the influence of clinicians whose video use would be high regardless of broadband access or who would prefer not to use video.

**Figure 1.**
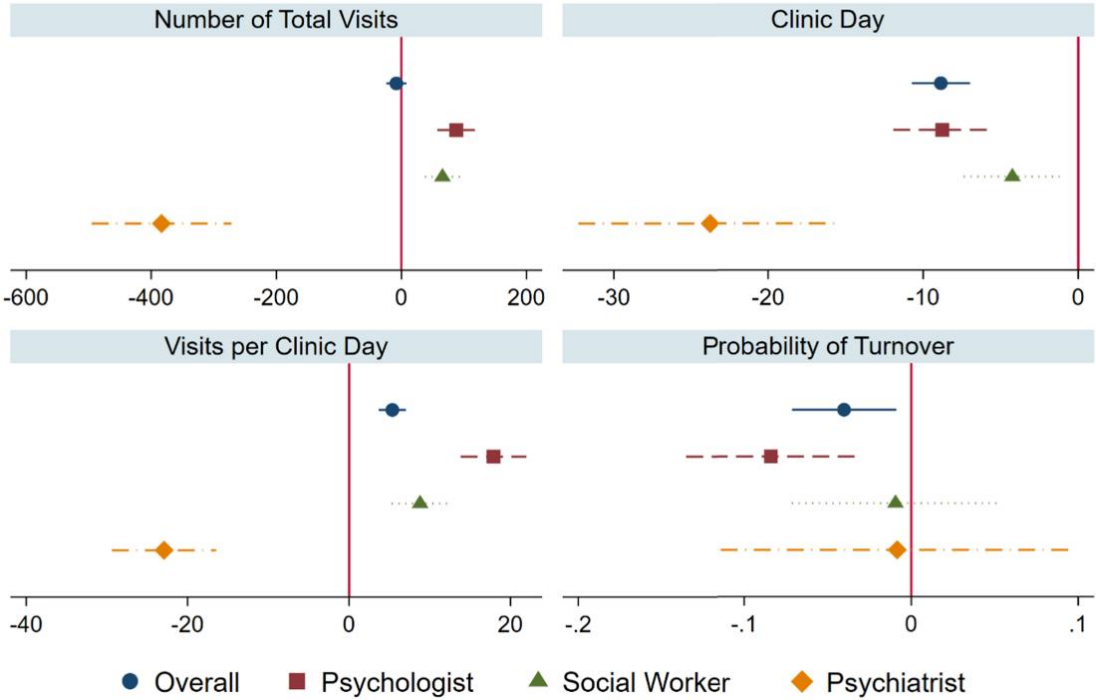
Clinician Video Visit Rates and Clinician Work Behavior Measures: All Clinicians and by Clinician Type. Forest plot of two-stage least squares estimates showing the association between the share of video visits (scaled 0-1) and four clinician work behavior outcomes (total visits, clinic days, visits per clinic day, and turnover probability). Estimates are presented overall and stratified by clinician type (psychologists, social workers, and psychiatrists/neurologists), with 95% confidence intervals. All models include region fixed effects, year fixed effects, and month fixed effects. Each coefficient represents the estimated change in the outcome associated with moving from 0 to 1 in the share of video visits (a complete shift from in-person to video visits). To rescale to a 10 percentage-point increase in video visit share, divide the coefficient by 10 (eg, 5.36/10 = 0.54 more visits per clinic day). Covariates include clinician characteristics (female, tenure, hourly wage), facility characteristics (RN turnover rate), and market characteristics (population, per capita income, unemployment rate (%), physicians, COVID-19 cases). First-stage *F*-statistics are 539.9 for the overall sample, 165.3 for psychologist, 187.9 for social worker, and 60.84 for psychiatrist. Full results are provided in Table S5. Confidence intervals are wider for psychiatrists because broadband explains less variation in video visit use for this group in the first stage than for psychologists and social workers.

The results show heterogeneity across clinician types in the changes in supply outcomes associated with increased video share (Figure 1; Table S5). For psychologists, a higher video share was associated with more total visits, fewer clinic days, more visits per clinic day, and lower turnover. For social workers, a higher video share was associated with more total visits, fewer clinic days, and more visits per clinic day, with no significant association with turnover. For psychiatrists, a higher video share was associated with fewer total visits, fewer clinic days, and fewer visits per clinic day, with no significant association with turnover.

Results were qualitatively similar across alternative samples and specifications (Table 3). Using an open clinician cohort that includes new entrants yielded consistent estimates for clinic days, visits per clinic day, and turnover (Panel B). Allowing for patient case-mix changes produced consistent estimates across 4 outcomes (Panel C). Relaxing the visit threshold to 1 visit per clinician-month yielded similar estimates, although turnover was no longer statistically significant (Panel D). Using the concurrent video share instead of the lagged video share yielded estimates in the same direction for clinic days, visits per clinic day, and turnover, with smaller magnitudes (Panel E). Total visits remained nonsignificant across all specifications.

**Table 3.** Alternative results on the relationship between clinician video visit rates and clinician work behavior measures.

	Number of total visits	Clinic days	Visits per clinic day	Probability of turnover
	Coeff (95% CI)	Coeff (95% CI)	Coeff (95% CI)	Coeff (95% CI)
Preferred model	−8.04	−8.86***	5.36***	−0.04*
	(−24.22, 8.14)	(−10.73, −6.98)	(3.68, 7.05)	(−0.07, −0.01)
Clinician cohort: Clinicians practicing during 2019-2021	11.37	−5.25***	4.14***	−0.03*
	(−2.47, 25.21)	(−6.78, −3.72)	(2.73, 5.55)	(−0.05, −0.01)
Patient cohort: Patients with any mental health visits during 2019-2021	−2.66	−6.91***	3.95***	−0.04*
	(−17.07, 11.75)	(−8.54, −5.28)	(2.50, 5.40)	(−0.06, −0.02)
Alternative threshold: ≥1 visit per clinician-month	−3.79	−8.76***	5.09***	−0.03
	(−22.45, 14.87)	(−10.97, −6.55)	(3.15, 7.03)	(−0.07, 0.01)
Endogenous variable: Concurrent video visit share	4.70	−5.15***	3.34***	−0.03*
	(−8.53, 17.93)	(−6.62, −3.68)	(1.95, 4.73)	(−0.05, −0.01)

^***^ *P* < 0.001, ** *P* < 0.01, * *P* < 0.05. Each coefficient represents the estimated change in the outcome associated with moving from 0 to 1 in the share of video visits (a complete shift from in-person to video visits). To rescale to a 10% point increase in video visit share, divide the coefficient by 10 (eg, 5.36/10 = 0.54 more visits per clinic day). All models include region fixed effects, year and month fixed effects, and the full covariate set. Panel A: preferred model (closed clinician cohort, 2019 patient cohort, and minimum 5 visits per clinician-month). Panels B through D each modify 1 element of the preferred model while holding all others constant: Panel B uses an open clinician cohort (all clinicians practicing during 2019-2021), Panel C uses an open patient cohort (patients with any mental health visits during 2019-2021), and Panel D uses a minimum of 1 visit per clinician-month. Panel E uses concurrent (unlagged) video visit share. First-stage *F*-statistics are 539.9 (Panel A), 678.7 (Panel B), 668.8 (Panel C), 416.7 (Panel D), and 781.0 (Panel E).

Table 4 presents characteristics of compliers, clinicians whose video visit rates are higher when their patients have better broadband access. Compared to the overall sample, compliers had fewer years since graduation (16.9 vs 18.5 years; *P* < 0.001), lower hourly wages ($98.6 vs $104.3; *P* < 0.001), and were located in markets with larger populations (7.5 vs 6.4 per 100 000; *P* < 0.001) and more physicians (0.20 vs 0.17 per 100 000; *P* < 0.001).

**Table 4.** Characteristics of clinicians affected by broadband availability.

		Overall mean	Complier mean	95% CI	*P* value
Clinician	Tenure years since graduation (year)	18.45	16.90	(16.50, 17.30)	<0.001
	Female (0-1)	0.63	0.64	(0.63, 0.66)	0.005
	Hourly wage (dollar)	104.32	98.56	(96.19, 100.93)	<0.001
Facility	RN turnover rate (0-1)	0.04	0.05	(0.05, 0.06)	<0.001
Market	Population (in 100 000 s)	6.36	7.45	(7.15, 7.75)	<0.001
	Per capita income (dollar)	54 445	53 878	(53,337, 54,420)	<0.001
	Physicians (in 100 000 s)	0.17	0.20	(0.19, 0.21)	<0.001
	Unemployment rate (%)	5.40	5.31	(5.22, 5.39)	<0.001
	COVID-19 cases (in 100 000 s)	0.10	0.25	(0.24, 0.26)	<0.001

The instrumental variable estimates in Table 2 and Figure 1 pertain to compliers, clinicians whose video visit use is higher than that of other clinicians due to their patients’ broadband availability being higher. The Complier Means in the table are the result of Abadie's kappa weighting method. Standard errors were obtained from 1000 bootstrap replications.

## Discussion

Mental health clinicians with higher proportions of telehealth visits showed no difference in total visits delivered (supply), but fewer clinic days (input), higher visit rates (throughput), and a lower probability of leaving clinical practice (retention). The findings were broadly consistent across psychologists and social workers, while psychiatrists showed distinct patterns. This study provides one of the first estimates of how telehealth relates to overall visit supply and the underlying supply components (input, throughput, retention). This study further contributes to the literature by documenting heterogeneous results across clinician types.

OLS and 2SLS estimates capture different aspects of the association between video use and clinician work behavior because they rely on different sources of variation. OLS reflects variation from all sources, including clinician characteristics correlated with both video adoption and supply outcomes, whereas 2SLS isolates variation in video use explained by patients’ broadband availability. OLS shows a positive association between video share and clinic days, potentially because clinicians whose video use is high for other reasons also tend to work more days. In contrast, 2SLS identifies clinicians whose video use responds to broadband access, among whom higher video use is associated with fewer clinic days. Among psychiatrists, broadband explained less first-stage variation in video use than for psychologists and social workers (Table S4, Panel C), which results in less precise estimates.

Our 2SLS estimates suggest that telehealth may reshape how clinicians work rather than how much care they deliver. Higher visits per clinic day are consistent with prior evidence of increased productivity following telehealth adoption,^17^ potentially through fewer no-shows^15^ and reduced time between visits from more efficient scheduling and clinical workflow steps (eg, patient intake and preparation). Fewer clinic days, combined with higher throughput, may indicate schedule compression, although our measure captures only the span between the first and last encounter and not how time between encounters was used. Lower turnover extends prior evidence on self-reported burnout associated with telehealth use.^18^ The null result for total visits may partly reflect the VA's salaried setting, where clinicians lack volume-based incentives. It may also reflect the inclusion of psychiatrists, among whom a smaller shoare of clinicians’ video use responds to broadband, and estimates are less precise. Because our estimates reflect clinician-month behavior, system-level supply may differ. The number of unique clinicians decreased over the study period (Figure S5).

Results differed across clinician types, potentially reflecting differences in service delivery and in the sensitivity of clinicians’ video use to patients’ broadband availability. Psychologists and social workers, who primarily provide psychotherapy, showed higher visits per clinic day, fewer clinic days, and more total visits. Because psychotherapy sessions are less easily shortened, telehealth gains for these clinicians may reflect reduced time between visits through more efficient scheduling and clinical workflow steps such as patient intake and preparation. Psychiatrists, whose practice more often involves shorter medication management visits, showed a different pattern. Their video use may also be less responsive to broadband availability if adoption is driven more by clinician preferences than by patient-side factors.

Our findings have implications for mental health workforce planning and telehealth policy. First, in addition to expanding patient access, telehealth may support clinician retention amid workforce shortages and rising attrition.^26-28^ If telehealth changes how clinicians allocate work and remain in practice, national workforce projections assuming fixed attrition rates may warrant reconsideration.^4^ Although total visits remained similar in our salaried setting, interstate licensing could allow clinicians to serve shortage areas remotely. Second, telehealth may improve throughput and reduce turnover, providing clinician-side evidence supporting permanent adoption. The recent addition of psychological testing to the federal government's permanent telehealth list aligns with our findings showing consistent positive associations between video visits and supply among psychologists. Given heterogeneous results across clinician types, telehealth investments may be particularly effective for psychologists and social workers, while implications for psychiatrists require further study.

## Conclusion

Our study provides new evidence on how telehealth provision relates to clinician supply. Overall, higher video visit shares were associated with more visits per clinic day, fewer clinic days, and lower turnover, with no significant change in total visits. There was heterogeneity across clinician types. Policymakers and managers can use these findings to better leverage telehealth in addressing mental health supply concerns.

## Supplementary Material

qxag070_Supplementary_Data
