# Supplementary material for: Telehealth and the work behavior of mental health clinicians
Source: Health Aff Sch. 2026 Mar 25;4(4):qxag070. doi: 10.1093/haschl/qxag070 (PMC13093905; doi:10.1093/haschl/qxag070)
Supplement: qxag070_Supplementary_Data [file qxag070_supplementary_data.zip › Song_Supplementary-material_2026.3.22..pdf]

Supplementary material

eMethod. Construction of instruments using broadband data

eTable 1. First-stage models: Association of each instrument (Fiber-25, Fiber-100, Fiber-250) on an endogenous variable (video visit rate)

eTable 2. Cross-sectional covariate balance grouped by observed video visit provision and by broadband coverage

eTable 3. Association of the instrument (internet availability) on an endogenous variable (video visit rate) and three of its components

eTable 4. First-stage results across alternative specifications

eTable 5. Full results of the Two-Stage Least Squares models

eTable 6a-d. Ordinary least squares and two-stage least squares estimates for four outcomes across alternative specifications

eFigure 1. Temporal variation of broadband availability across speed by year

eFigure 2. Distribution of broadband availability among clinicians by fiber speed

eFigure 3. Month-to-month change in broadband availability

eFigure 4. Unadjusted total visits, clinic day, visits per clinic day, and turnover, stratified by clinician types

eFigure 5. Trend of the number of clinicians

References

eMethod. Construction of instruments using broadband data

We constructed instruments using broadband availability from the Federal Communications Commission (FCC) to instrument telehealth provision. Prior studies have used broadband availability as an instrument in other settings, such as the diffusion of misinformation[1] and the effects of online health information[2], but its application in the telehealth context remains limited.

FCC Data. The FCC reports broadband availability, rather than actual use, at the county level. The data include the proportion of the population with access to different internet types (fiber, asymmetric digital subscriber line, cable, satellite), a range of internet speeds (from 0.2 to 1000 Mbps), and the number of internet service providers (ISPs) offering a given internet type and speed (0, 1, 2, or 3 or more ISPs). For example, FCC data indicates that 41 percent of Suffolk County residents have access to at least one ISP providing 250 Mbps fiber internet.

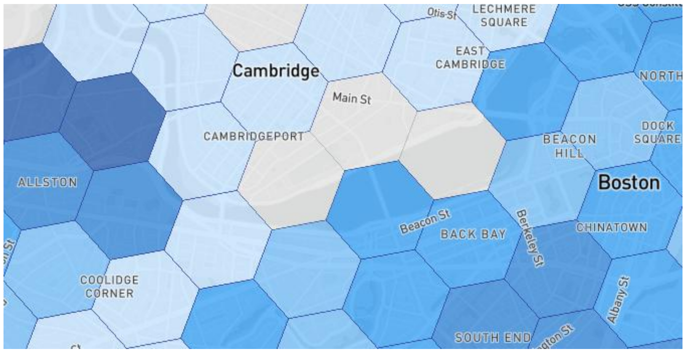

41% of Suffolk County residents have access to at least one ISP providing 250 Mbps fiber internet.

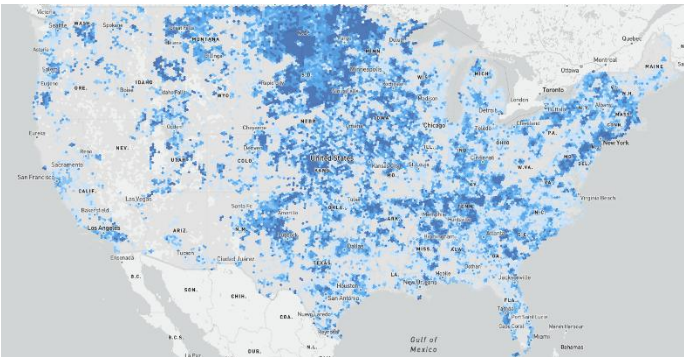

National county-level variation of 250 Mbps fiber internet (the widest variation among certain internet types and speeds)

Instrument Construction. For each clinician-month, we calculated the average broadband availability among all patient visits to that clinician during the month. Each patient visit contributed to the weighted average according to visit frequency, such that patients with multiple visits within a month exerted proportionally greater influence on the broadband measure than those with a single visit. When a patient saw multiple clinicians, their broadband data were incorporated into the calculations for each clinician they visited. This design ensures that the broadband variable reflects actual utilization patterns and the local access environment experienced by each clinician on a monthly basis.

We tested several specifications to identify the broadband measure that provided sufficient variation and theoretical justification for use as an instrument. Broadband availability for any internet type with 250 Mbps speed produced wide county-level variation but yielded a left-skewed distribution when aggregated to the clinician level, indicating limited variation (Panel A). In contrast, the percentage of county residents with access to fiber internet at 250 Mbps displayed greater cross-county variation and a near-normal distribution after aggregation (Panel B). Conceptually, fiber infrastructure is costlier and more difficult to deploy than other technologies, making it less responsive to short-term federal broadband expansion initiatives introduced during the COVID-19 pandemic (e.g., the Capital Projects Fund). For these empirical and conceptual reasons, the 250 Mbps fiber availability measure was selected as the preferred specification.

We also examined availability of fiber internet at 1000 Mbps, the maximum speed reported by the FCC. However, most counties had zero population coverage at this speed, resulting in a highly right-skewed distribution at both the county and clinician levels (Panel C). Given the limited variation observed between 2019 and 2021, this specification was not retained, though it may become more suitable as ultra-high-speed broadband expands in future years.

In summary, the preferred instrument, the share of county residents with access to 250 Mbps fiber internet, provided the strongest empirical variation and conceptual validity for capturing local broadband capacity relevant to telehealth provision.

	Panel A: Any technology type, 250 Mbps	Panel B: Fiber type, 250 Mbps (preferred)	Panel C: Fiber type, 1000 Mbps
County-level variation	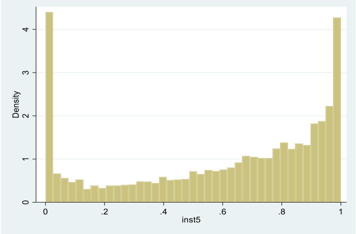	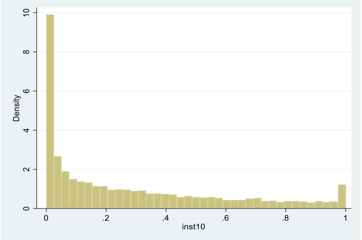	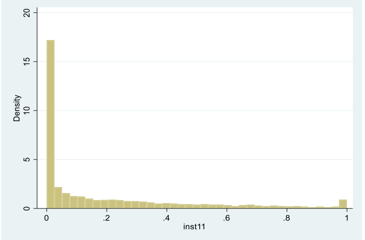
Clinician-level variation	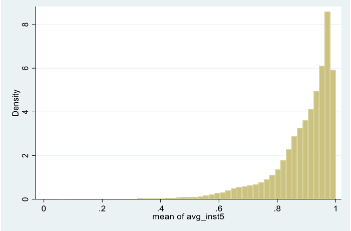	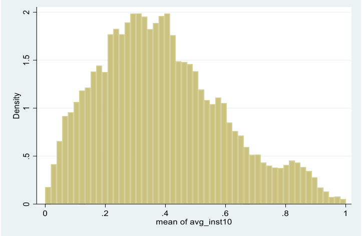	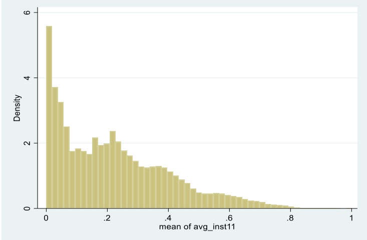

eTable 1. First-stage models: Association of each instrument (Fiber-25, Fiber-100, Fiber-250) on an endogenous variable (video visit rate)

	Endogenous variable: Video Share (%, 3-month lag)		
Instrument 1: Fiber-25	0.04***		
	(0.00)		
Instrument 2: Fiber-100		0.04***	
		(0.00)	
Instrument 3: Fiber-250 (preferred)			0.06***
			(0.00)
Tenure year since graduation (year)	-0.00***	-0.00***	-0.00***
	(0.00)	(0.00)	(0.00)
Female (0-1)	0.03***	0.03***	0.03***
	(0.00)	(0.00)	(0.00)
Hourly wage (dollar)	-0.00***	-0.00***	-0.00***
	(0.00)	(0.00)	(0.00)
RN turnover rate (0-1)	-0.04***	-0.04***	-0.04***
	(0.01)	(0.01)	(0.01)
Population (in 100,000s)	-0.00***	-0.00***	-0.00***
	(0.00)	(0.00)	(0.00)
Per capita income (dollar)	0.00***	0.00***	0.00***
	(0.00)	(0.00)	(0.00)
Physicians (in 100,000s)	0.05***	0.05***	0.05***
	(0.00)	(0.00)	(0.00)
Unemployment rate (%)	0.01***	0.01***	0.01***
	(0.00)	(0.00)	(0.00)
COVID-19 cases (in 100,000s)	0.12***	0.12***	0.12***
	(0.00)	(0.00)	(0.00)
Constant	0.13***	0.13***	0.13***
	(0.00)	(0.00)	(0.00)
Region fixed effects	X	X	X
Year fixed effects	X	X	X
Month fixed effects	X	X	X
Observations	226,153	226,153	226,153
R-squared	0.38	0.38	0.38
First-stage F-statistics	225.2	263.3	539.9

Note: *** p<0.001, ** p<0.01, * p<0.05. This table reports the first-stage association between three broadband availability instruments (Fiber-25, Fiber-100, Fiber-250 Mbps) and video visit share, demonstrating instrument relevance across alternative speed thresholds. All models include region fixed effects, year fixed effects, month fixed effects, and the full covariate set. Huber-White robust standard errors are reported.

eTable 2. Cross-sectional covariate balance grouped by observed video visit provision and by broadband coverage

	**A. Provision of video visits among outpatient mental health visits**					**B. Internet broadband coverage among clinicians' patients (IV)**				
	Below median<br><br>N=113,101		Above median<br><br>N=113,052			Below median (poor coverage)<br>N=113,077		Above median<br><br>N=113,076		
	Mean	(SD)	Mean	(SD)	Standardized difference	Mean	(SD)	Mean	(SD)	Standardized difference
Tenure year since graduation (year)	18.90	(10.71)	17.99	(9.55)	-0.06	18.37	(10.05)	18.52	(10.26)	0.01
Female (0-1)	0.61	(0.49)	0.66	(0.47)	0.09	0.63	(0.48)	0.64	(0.48)	0.01
Hourly wage (dollar)	105.31	(63.21)	103.32	(58.04)	-0.02	101.43	(60.47)	107.21	(60.76)	0.07
RN turnover rate (0-1)	0.04	(0.09)	0.04	(0.09)	-0.01	0.04	(0.08)	0.05	(0.10)	0.05
Population (in 100,000s)	6.55	(7.63)	6.17	(7.34)	-0.04	4.80	(6.93)	7.92	(7.70)	0.30
Per capita income (dollar)	52,816	(13,212)	56,074	(13,328)	0.17	50,232	(11,931)	58,657	(13,403)	0.47
Physicians (in 100,000s)	0.17	(0.23)	0.17	(0.22)	0.01	0.13	(0.22)	0.20	(0.23)	0.23
Unemployment rate (%)	4.63	(2.04)	6.16	(2.18)	0.51	5.30	(2.21)	5.49	(2.27)	0.06
COVID-19 cases (in 100,000s)	0.03	(0.11)	0.17	(0.26)	0.53	0.07	(0.15)	0.13	(0.25)	0.23

Note: This table compares clinician, facility, and market characteristics between groups defined by broadband availability (Panel B) and by video visit provision (Panel A) to assess the plausibility of the exclusion restriction. Standardized differences are reported to quantify covariate imbalance across groups.

eTable 3. Association of the instrument (internet availability) on an endogenous variable (video visit rate) and three of its components

	Endogenous variable	Components of the endogenous variable		
	Video visit rate	Number of video visits	Number of phone visits	Number of in-person visits
Fiber-250	0.06***	6.17***	-2.46***	-4.65***
	(0.00)	(0.24)	(0.28)	(0.39)
Tenure year since graduation (year)	-0.00***	-0.14***	0.10***	0.20***
	(0.00)	(0.00)	(0.01)	(0.01)
Female (0-1)	0.03***	1.89***	-0.89***	-4.85***
	(0.00)	(0.09)	(0.11)	(0.15)
Hourly wage (dollar)	-0.00***	-0.01***	0.07***	0.03***
	(0.00)	(0.00)	(0.00)	(0.00)
RN turnover rate (0-1)	-0.04***	-1.82***	2.65***	2.60***
	(0.01)	(0.50)	(0.57)	(0.77)
Population (in 100,000s)	-0.00***	-0.11***	-0.11***	0.20***
	(0.00)	(0.01)	(0.01)	(0.02)
Per capita income (dollar)	0.00***	0.00***	-0.00	-0.00***
	(0.00)	(0.00)	(0.00)	(0.00)
Physicians (in 100,000s)	0.05***	4.75***	1.90***	5.41***
	(0.00)	(0.32)	(0.38)	(0.57)
Unemployment rate (%)	0.01***	2.10***	3.47***	-5.25***
	(0.00)	(0.03)	(0.04)	(0.04)
COVID-19 cases (in 100,000s)	0.12***	10.85***	5.60***	-10.98***
	(0.00)	(0.40)	(0.39)	(0.34)
Constant	0.13***	3.15***	-5.11***	66.89***
	(0.00)	(0.33)	(0.41)	(0.49)
Region fixed effects	X	X	X	X
Year fixed effects	X	X	X	X
Month fixed effects	X	X	X	X
Observations	226,153	226,153	226,153	226,153
R-squared	0.38	0.33	0.28	0.38

Note: *** p<0.001, ** p<0.01, * p<0.05. This table reports the first-stage associations between the broadband instrument and visit components (video, phone, in-person). All models include region fixed effects, year fixed effects, month fixed effects, and the full covariate set. Huber-White robust standard errors are reported.

eTable 4. First-stage results across alternative specifications

	Sample	Specification				Outcome: % Video Visit (3-month lag)				
		Cross-Sectional FE	Time FE	All covariates	Additional Time-varying Covariates	Coef	SE	Observations	R-squared	First-stage F
**Panel A: 18 Region-Level Fixed Effects with Additional Time-Varying County-level Covariates**										
(1)	Overall	Region	Year, Month	X	-	0.07***	(0.00)	226,153	0.38	744.2
(2)	Overall	Region	Year, Month	X	Per capita income	0.06***	(0.00)	226,153	0.38	539.9
(3)	Overall	Region	Year, Month	X	Area Deprivation Index	0.04***	(0.00)	226,153	0.38	161.3
(4)	Overall	Region	Year, Month	X	# Visits (4-month lag)	0.09***	(0.00)	211,611	0.39	2443
**Panel B: Facility and Clinician Fixed Effects**										
(5)	Overall	Facility	Year, Month	X	-	-0.00	(0.00)	226,153	0.42	1.1
(6)	Overall	Clinician	Year, Month	X	-	0.01	(0.01)	225,722	0.68	3.0
(7)	Overall	Facility	Year, Month	X	# Visits (4-month lag)	-0.00	(0.00)	211,611	0.42	0.3
**Panel C: Subgroup Analyses**										
(8)	Psychologist	Region	Year, Month	X	Per capita income	0.06***	(0.00)	87,605	0.45	165.3
(9)	Social worker	Region	Year, Month	X	Per capita income	0.06***	(0.00)	75,683	0.40	187.9
(10)	Psychiatrist	Region	Year, Month	X	Per capita income	0.03***	(0.00)	62,865	0.31	60.8

Note: *** p<0.001, ** p<0.01, * p<0.05. This table presents a specification gradient for the first-stage regression, comparing alternative specifications. Huber-White robust standard errors are reported.

eTable 5. Full results of the Two-Stage Least Squares models

Sample	Overall sample				Psychologist			
Outcomes	Number of total visits	Clinic days	Visits per clinic day	Probability of turnover	Number of total visits	Clinic days	Visits per clinic day	Probability of turnover
Video visit rate (0-1)	-8.04	-8.86***	5.36***	-0.04*	87.74***	-8.76***	17.90***	-0.08**
	(8.25)	(0.96)	(0.86)	(0.02)	(15.29)	(1.61)	(2.08)	(0.03)
Female (0-1)	0.16***	-0.01*	0.03***	0.00	0.33***	-0.02***	0.07***	-0.00
	(0.02)	(0.00)	(0.00)	(0.00)	(0.05)	(0.00)	(0.01)	(0.00)
Tenure year since graduation (year)	-4.02***	-0.01	-0.31***	0.00	-8.65***	-0.18**	-0.75***	0.00
	(0.33)	(0.04)	(0.04)	(0.00)	(0.64)	(0.07)	(0.09)	(0.00)
Hourly wage (dollar)	0.09***	-0.00***	0.01***	-0.00***	-0.15***	-0.01***	-0.01***	0.00**
	(0.00)	(0.00)	(0.00)	(0.00)	(0.01)	(0.00)	(0.00)	(0.00)
RN turnover rate (0-1)	3.67***	-0.22	0.52***	-0.01**	9.15***	-0.15	1.36***	-0.01*
	(1.09)	(0.12)	(0.11)	(0.00)	(1.95)	(0.20)	(0.26)	(0.00)
Population (in 100,000s)	-0.03	-0.03***	0.03***	-0.00	0.37***	-0.03***	0.08***	-0.00
	(0.02)	(0.00)	(0.00)	(0.00)	(0.05)	(0.01)	(0.01)	(0.00)
Per capita income (dollar)	-0.00***	-0.00***	0.00***	0.00*	-0.00***	-0.00*	-0.00	0.00*
	(0.00)	(0.00)	(0.00)	(0.00)	(0.00)	(0.00)	(0.00)	(0.00)
Physicians (in 100,000s)	12.80***	1.06***	0.24**	-0.00	5.01**	0.57**	0.04	0.00
	(0.79)	(0.09)	(0.09)	(0.00)	(1.85)	(0.18)	(0.26)	(0.00)
Unemployment rate (%)	0.64***	0.25***	-0.16***	0.00	-1.65***	0.35***	-0.53***	0.00*
	(0.13)	(0.02)	(0.01)	(0.00)	(0.38)	(0.04)	(0.05)	(0.00)
COVID-19 cases (in 100,000s)	7.68***	1.66***	-0.51***	0.00	-7.16**	1.57***	-2.28***	0.01*
	(1.18)	(0.14)	(0.12)	(0.00)	(2.31)	(0.25)	(0.31)	(0.00)
Observations	226,153	226,153	226,153	226,153	87,605	87,605	87,605	87,605
First-stage F-statistics	539.9	539.9	539.9	539.9	165.3	165.3	165.3	165.3

Note: *** p<0.001, ** p<0.01, * p<0.05. This table reports full Two-Stage Least Squares results with all covariate coefficients for the overall sample and three clinician subgroups across four outcomes. Video visit share is measured on a 0-1 scale. First-stage F statistics are the Kleibergen-Paap rk Wald F statistics. Huber-White robust standard errors are reported.

Sample	Social worker				Psychiatrist			
Outcomes	Number of total visits	Clinic days	Visits per clinic day	Probability of turnover	Number of total visits	Clinic days	Visits per clinic day	Probability of turnover
Video visit rate (0-1)	65.75***	-4.25**	8.80***	-0.01	-383.42***	-23.79***	-22.91***	-0.01
	(14.27)	(1.59)	(1.79)	(0.03)	(57.03)	(4.34)	(3.30)	(0.05)
Female (0-1)	0.24***	0.02***	0.01	0.00	-0.30***	-0.02***	-0.01***	0.00
	(0.03)	(0.00)	(0.00)	(0.00)	(0.06)	(0.00)	(0.00)	(0.00)
Tenure year since graduation (year)	-4.52***	0.06	-0.56***	0.00	10.96***	0.65***	0.68***	0.00
	(0.53)	(0.06)	(0.07)	(0.00)	(1.96)	(0.15)	(0.11)	(0.00)
Hourly wage (dollar)	-0.08***	-0.01***	0.00*	-0.00	-0.09***	-0.01***	0.00***	-0.00**
	(0.01)	(0.00)	(0.00)	(0.00)	(0.01)	(0.00)	(0.00)	(0.00)
RN turnover rate (0-1)	8.73***	0.10	0.48*	-0.00	-3.64	-0.38	-0.18	-0.01
	(1.81)	(0.20)	(0.23)	(0.00)	(3.98)	(0.30)	(0.23)	(0.00)
Population (in 100,000s)	0.03	-0.03***	0.03***	0.00	-0.75***	-0.05***	-0.03***	-0.00
	(0.03)	(0.00)	(0.00)	(0.00)	(0.09)	(0.01)	(0.01)	(0.00)
Per capita income (dollar)	-0.00***	-0.00***	0.00***	-0.00	0.00***	0.00***	0.00*	0.00
	(0.00)	(0.00)	(0.00)	(0.00)	(0.00)	(0.00)	(0.00)	(0.00)
Physicians (in 100,000s)	15.66***	1.90***	-0.53**	-0.00	11.53***	0.54***	0.62***	-0.00
	(1.41)	(0.14)	(0.19)	(0.00)	(2.13)	(0.16)	(0.12)	(0.00)
Unemployment rate (%)	-0.95***	0.14***	-0.24***	-0.00	2.97***	0.24***	0.09***	-0.00
	(0.19)	(0.02)	(0.03)	(0.00)	(0.30)	(0.02)	(0.02)	(0.00)
COVID-19 cases (in 100,000s)	-7.18*	1.62***	-1.78***	-0.00	29.40***	1.81***	1.67***	0.00
	(3.04)	(0.34)	(0.38)	(0.01)	(4.43)	(0.33)	(0.25)	(0.00)
Observations	75,683	75,683	75,683	75,683	62,865	62,865	62,865	62,865
First-stage F-statistics	187.9	187.9	187.9	187.9	60.84	60.84	60.84	60.84

eTable 6a. Ordinary least squares and two-stage least squares estimates for four outcomes across alternative specifications

	Sample	Specification				Outcome: Number of Total Visits				
						OLS		2SLS		
		Cross-Sectional FE	Time FE	All covariates	Additional Time-varying Covariates	Coef	SE	Coef	SE	First-stage F
**Panel A: 18 Region-Level Fixed Effects with Additional Time-Varying County-level Covariates**										
(1)	Overall	Region	Year, Month	X	-	-9.24***	(0.39)	-18.90**	(7.14)	744.2
(2)	Overall	Region	Year, Month	X	Per capita income	-9.18***	(0.39)	-8.04	(8.25)	539.9
(3)	Overall	Region	Year, Month	X	Area Deprivation Index	-9.36***	(0.39)	-60.93***	(15.60)	161.3
(4)	Overall	Region	Year, Month	X	# Visits (4-month lag)	0.81***	(0.21)	-11.45**	(3.64)	777.2
**Panel B: Facility and Clinician Fixed Effects**										
(5)	Overall	Facility	Year, Month	X	-	-7.02***	(0.36)	369.48	(384.28)	1.1
(6)	Overall	Clinician	Year, Month	X	-	-1.40***	(0.27)	164.69	(113.01)	3.0
(7)	Overall	Facility	Year, Month	X	# Visits (4-month lag)	1.06***	(0.22)	773.15	(1594.86)	0.3
**Panel C: Subgroup Analyses**										
(8)	Psychologist	Region	Year, Month	X	Per capita income	-3.12***	(0.55)	87.74***	(15.29)	165.3
(9)	Social worker	Region	Year, Month	X	Per capita income	6.89***	(0.66)	65.75***	(14.27)	187.9
(10)	Psychiatrist	Region	Year, Month	X	Per capita income	-10.27***	(1.02)	-383.42***	(57.03)	60.8

Note: *** p<0.001, ** p<0.01, * p<0.05. This table presents a specification gradient comparing ten specifications for each outcome. Each sub-table reports OLS and 2SLS estimates along with the first-stage Kleibergen-Paap F statistic. Video visit share is measured on a 0-1 scale. Huber-White robust standard errors are reported.

eTable 6b. Ordinary least squares and two-stage least squares estimates for four outcomes across alternative specifications

	Sample	Specification				Outcome: Clinic Days				
						OLS		2SLS		
		Cross-Sectional FE	Time FE	All covariates	Additional Time-varying Covariates	Coef	SE	Coef	SE	First-stage F
**Panel A: 18 Region-Level Fixed Effects with Additional Time-Varying County-level Covariates**										
(1)	Overall	Region	Year, Month	X	-	0.14**	(0.05)	-10.67***	(0.85)	744.2
(2)	Overall	Region	Year, Month	X	Per capita income	0.16***	(0.05)	-8.86***	(0.96)	539.9
(3)	Overall	Region	Year, Month	X	Area Deprivation Index	0.15***	(0.05)	-22.87***	(2.41)	161.3
(4)	Overall	Region	Year, Month	X	Clinic days (4-month lag)	0.14***	(0.03)	-2.17***	(0.42)	762.6
**Panel B: Facility and Clinician Fixed Effects**										
(5)	Overall	Facility	Year, Month	X	-	0.09*	(0.04)	121.19	(115.12)	1.1
(6)	Overall	Clinician	Year, Month	X	-	-0.03	(0.03)	-9.95	(8.98)	3.0
(7)	Overall	Facility	Year, Month	X	Clinic days (4-month lag)	0.16***	(0.03)	123.86	(242.80)	0.3
**Panel C: Subgroup Analyses**										
(8)	Psychologist	Region	Year, Month	X	Per capita income	0.47***	(0.07)	-8.76***	(1.61)	165.3
(9)	Social worker	Region	Year, Month	X	Per capita income	1.55***	(0.08)	-4.25**	(1.59)	187.9
(10)	Psychiatrist	Region	Year, Month	X	Per capita income	-0.25*	(0.11)	-23.79***	(4.34)	60.8

eTable 6c. Ordinary least squares and two-stage least squares estimates for four outcomes across alternative specifications

	**Sample**	**Specification**				**Outcome: Visits per Clinic Day**				
						OLS		2SLS		
		Cross-Sectional FE	Time FE	All covariates	Additional Time-varying Covariates	Coef	SE	Coef	SE	First-stage F
**Panel A: 18 Region-Level Fixed Effects with Additional Time-Varying County-level Covariates**										
(1)	Overall	Region	Year, Month	X	-	-1.15***	(0.04)	6.89***	(0.79)	744.2
(2)	Overall	Region	Year, Month	X	Per capita income	-1.17***	(0.04)	5.36***	(0.86)	539.9
(3)	Overall	Region	Year, Month	X	Area Deprivation Index	-1.17***	(0.04)	13.84***	(1.91)	161.3
(4)	Overall	Region	Year, Month	X	Visit rate (4-month lag)	0.13***	(0.02)	-1.22***	(0.21)	2443
**Panel B: Facility and Clinician Fixed Effects**										
(5)	Overall	Facility	Year, Month	X	-	-0.95***	(0.04)	-60.98	(58.18)	1.1
(6)	Overall	Clinician	Year, Month	X	-	-0.15***	(0.03)	32.17	(20.25)	3.0
(7)	Overall	Facility	Year, Month	X	Visit rate (4-month lag)	-0.14***	(0.03)	-43.79	(78.58)	0.3
**Panel C: Subgroup Analyses**										
(8)	Psychologist	Region	Year, Month	X	Per capita income	-0.74***	(0.06)	17.90***	(2.08)	165.3
(9)	Social worker	Region	Year, Month	X	Per capita income	-0.81***	(0.09)	8.80***	(1.79)	187.9
(10)	Psychiatrist	Region	Year, Month	X	Per capita income	-0.76***	(0.05)	-22.91***	(3.30)	60.8

eTable 6d. Ordinary least squares and two-stage least squares estimates for four outcomes across alternative specifications

	Sample	Specification				Outcome: Probability of Turnover				
						OLS		2SLS		
		Cross-Sectional FE	Time FE	All covariates	Additional Time-varying Covariates	Coef	SE	Coef	SE	First-stage F
**Panel A: 18 Region-Level Fixed Effects with Additional Time-Varying County-level Covariates**										
(1)	Overall	Region	Year, Month	X	-	0.00	(0.00)	-0.03*	(0.01)	744.2
(2)	Overall	Region	Year, Month	X	Per capita income	0.00	(0.00)	-0.04*	(0.02)	539.9
(3)	Overall	Region	Year, Month	X	Area Deprivation Index	0.00	(0.00)	-0.07*	(0.03)	161.3
(4)	Overall	Region	Year, Month	X	Turnover (4-month lag)	0.00	(0.00)	-0.03*	(0.01)	766.6
**Panel B: Facility and Clinician Fixed Effects**										
(5)	Overall	Facility	Year, Month	X	-	0.00	(0.00)	0.09	(0.30)	1.1
(6)	Overall	Clinician	Year, Month	X	-	-0.00	(0.00)	0.01	(0.21)	3.0
(7)	Overall	Facility	Year, Month	X	Turnover (4-month lag)	0.00	(0.00)	0.05	(0.63)	0.3
**Panel C: Subgroup Analyses**										
(8)	Psychologist	Region	Year, Month	X	Per capita income	0.00	(0.00)	-0.08**	(0.03)	165.3
(9)	Social worker	Region	Year, Month	X	Per capita income	0.00	(0.00)	-0.01	(0.03)	187.9
(10)	Psychiatrist	Region	Year, Month	X	Per capita income	0.00	(0.00)	-0.01	(0.05)	60.8

eFigure 1. Temporal variation of broadband availability across speed by year

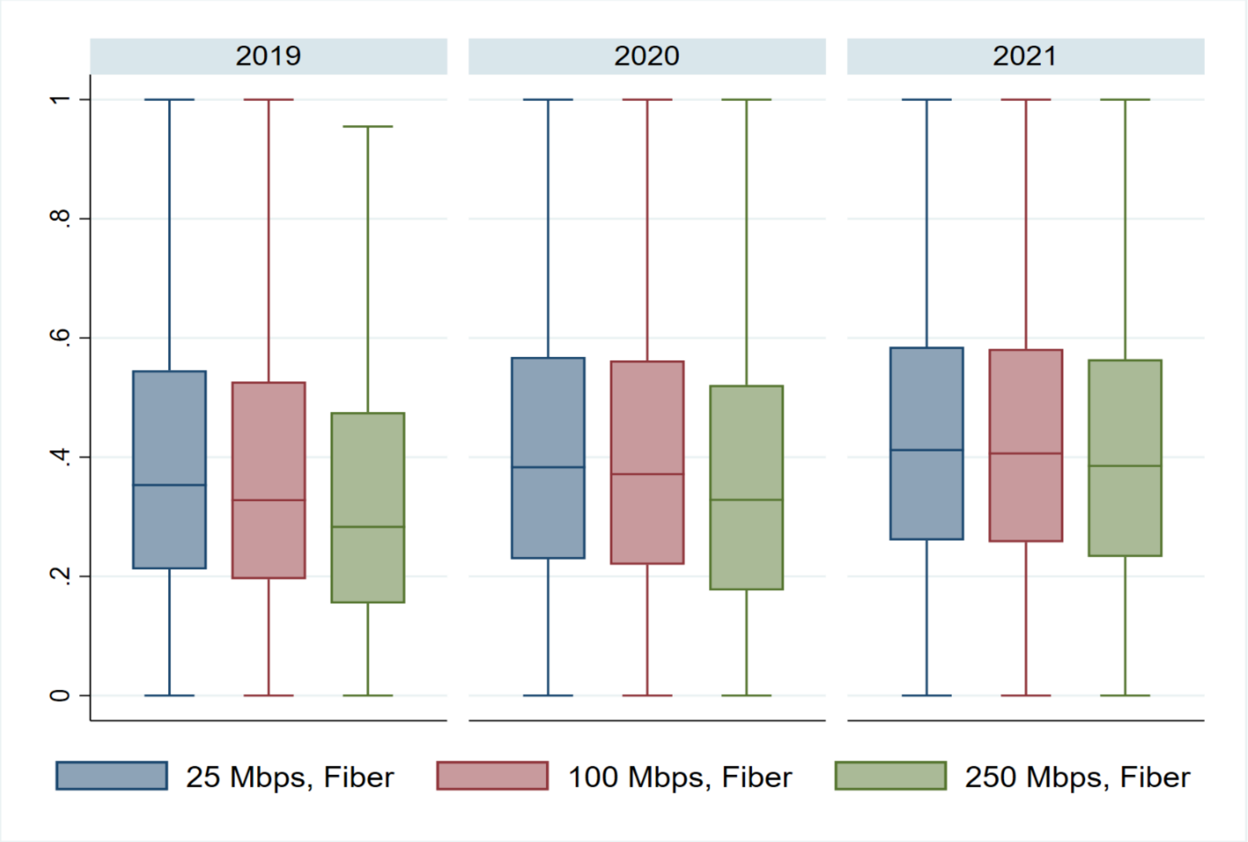

Note: This figure displays temporal variation in broadband availability across speed thresholds, demonstrating over-time variation for instrument relevance.

eFigure 2. Distribution of broadband availability among clinicians by fiber speed

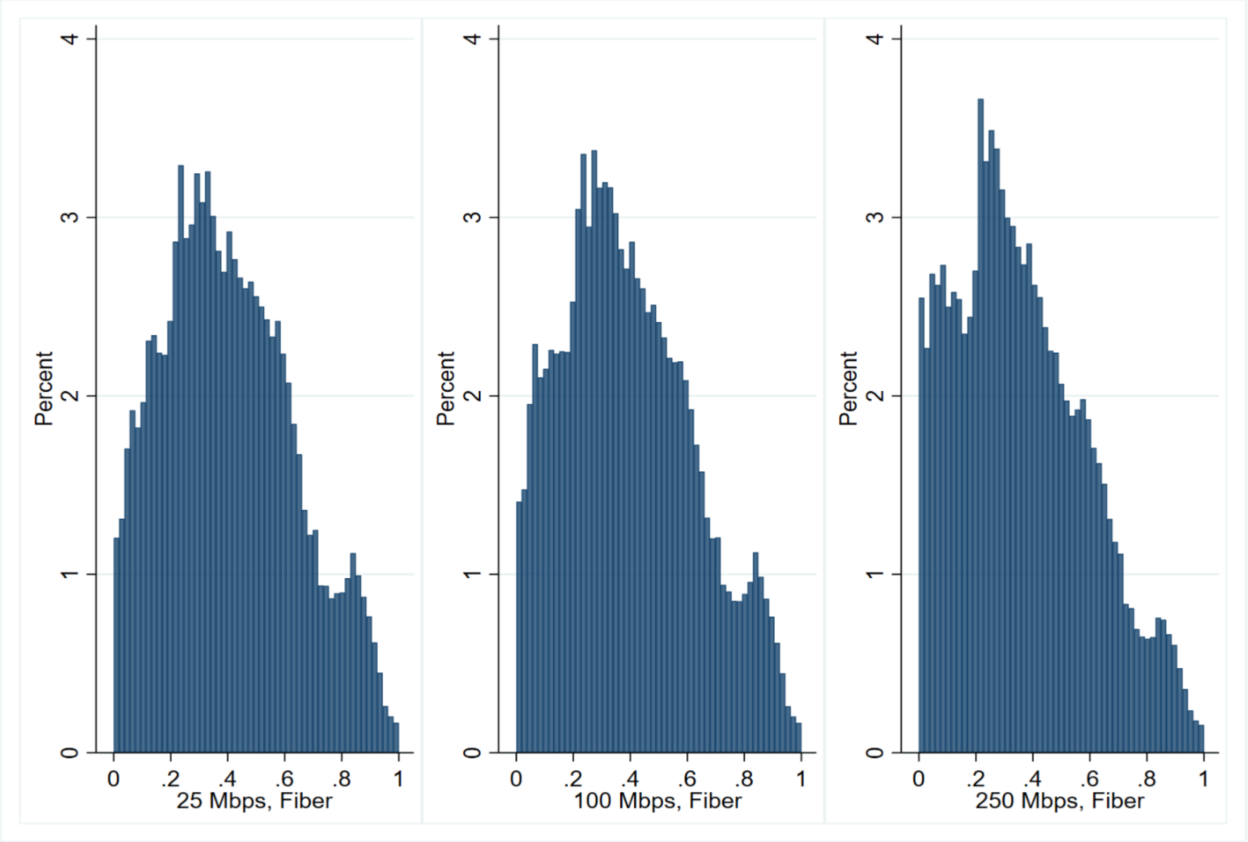

Note: This figure shows the distribution of the clinician-level broadband instrument, demonstrating near-normal distribution for the instrument specifications.

eFigure 3. Month-to-month change in broadband availability

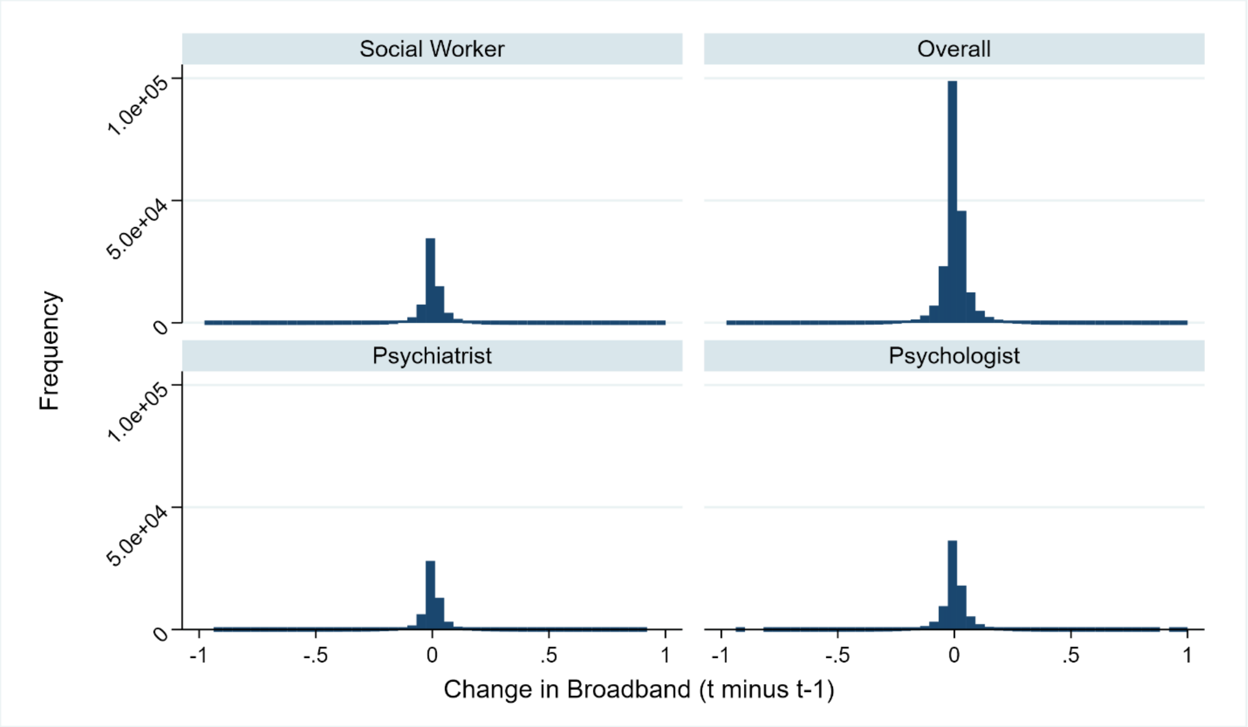

Note: Each panel plots the distribution of month-to-month changes in the broadband instrument (t minus t-1) at the clinician-month level.

eFigure 4. Unadjusted total visits, clinic day, visits per clinic day, and turnover, stratified by clinician types

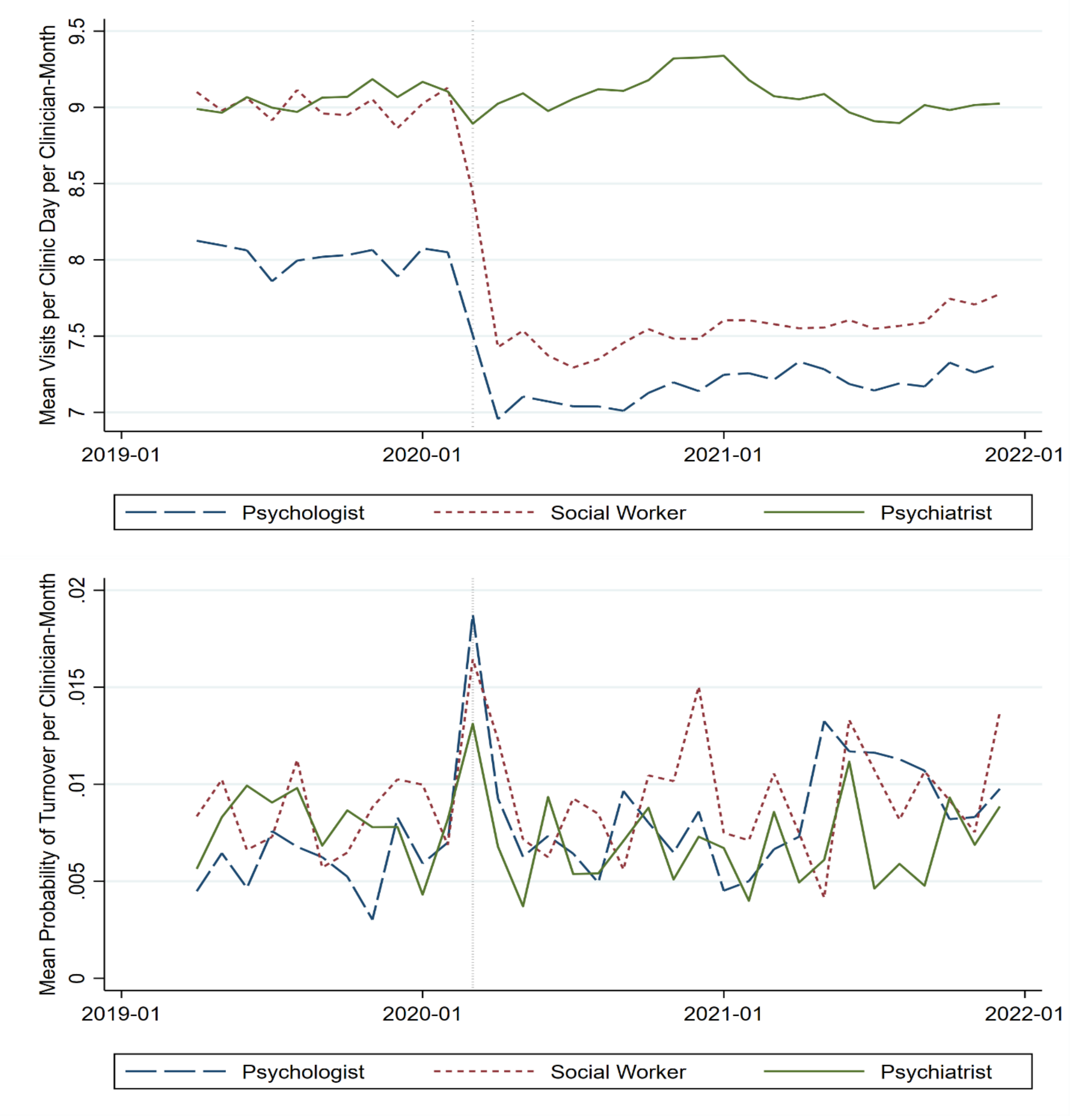

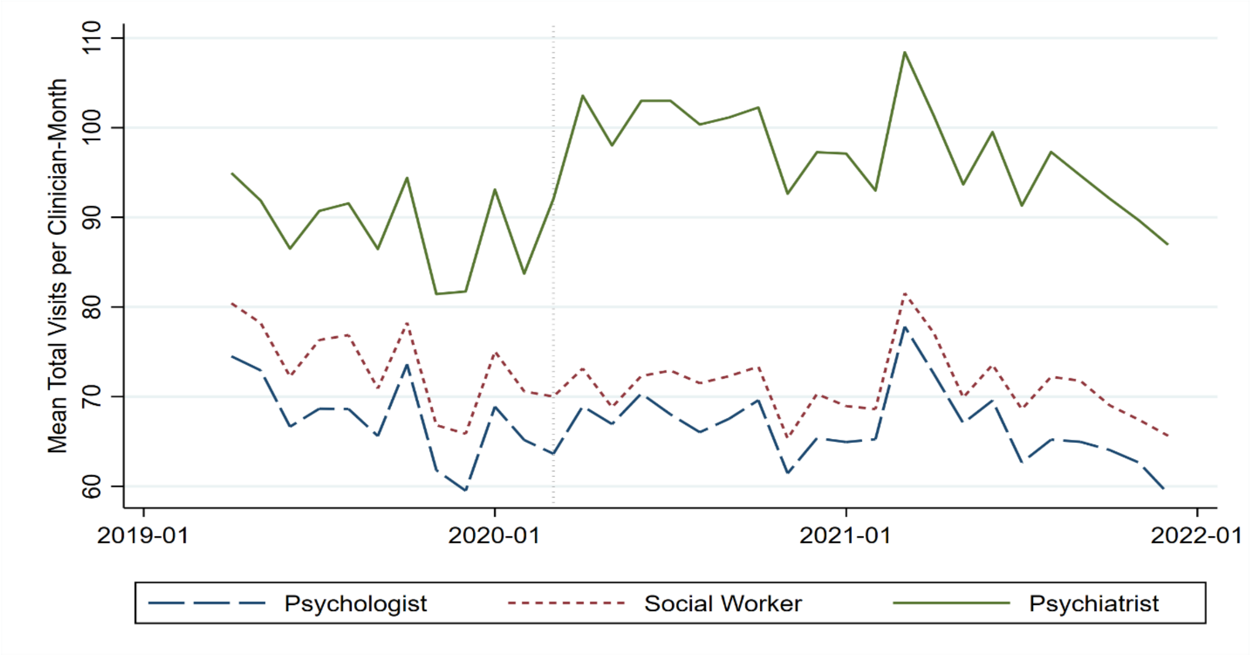

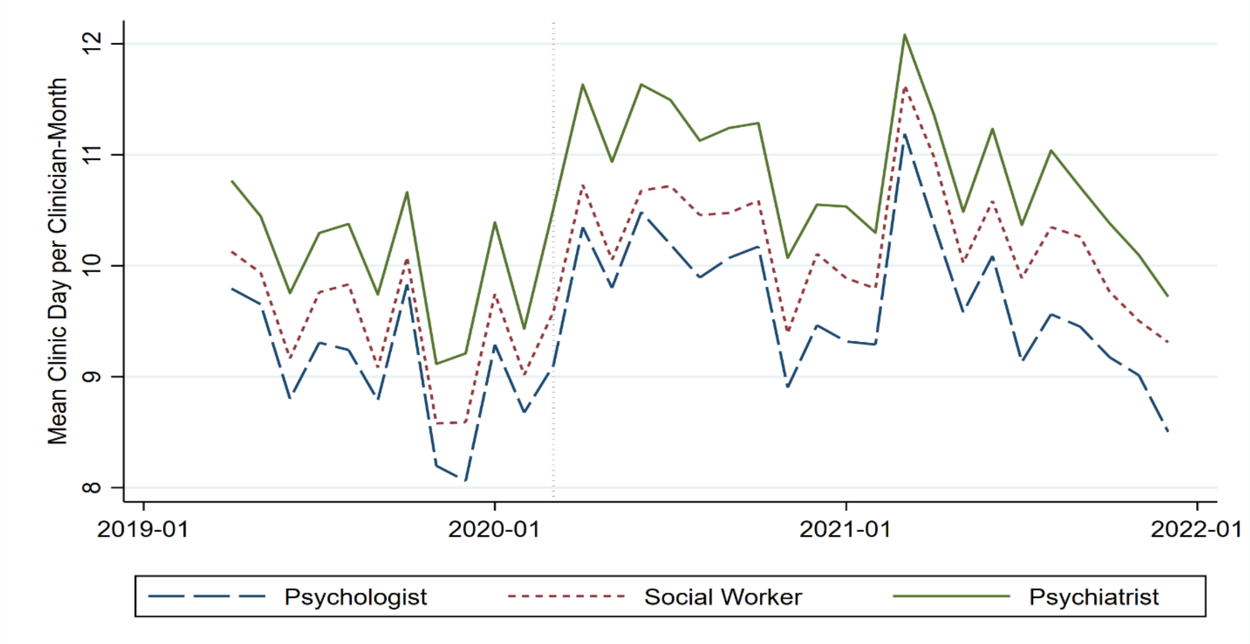

Note: These figures show temporal trends in total visits, clinic day, visits per clinic day, and turnover for the closed clinician cohort, providing descriptive context. These are descriptive trends, not causal estimates.

eFigure 5. Trend of the number of clinicians

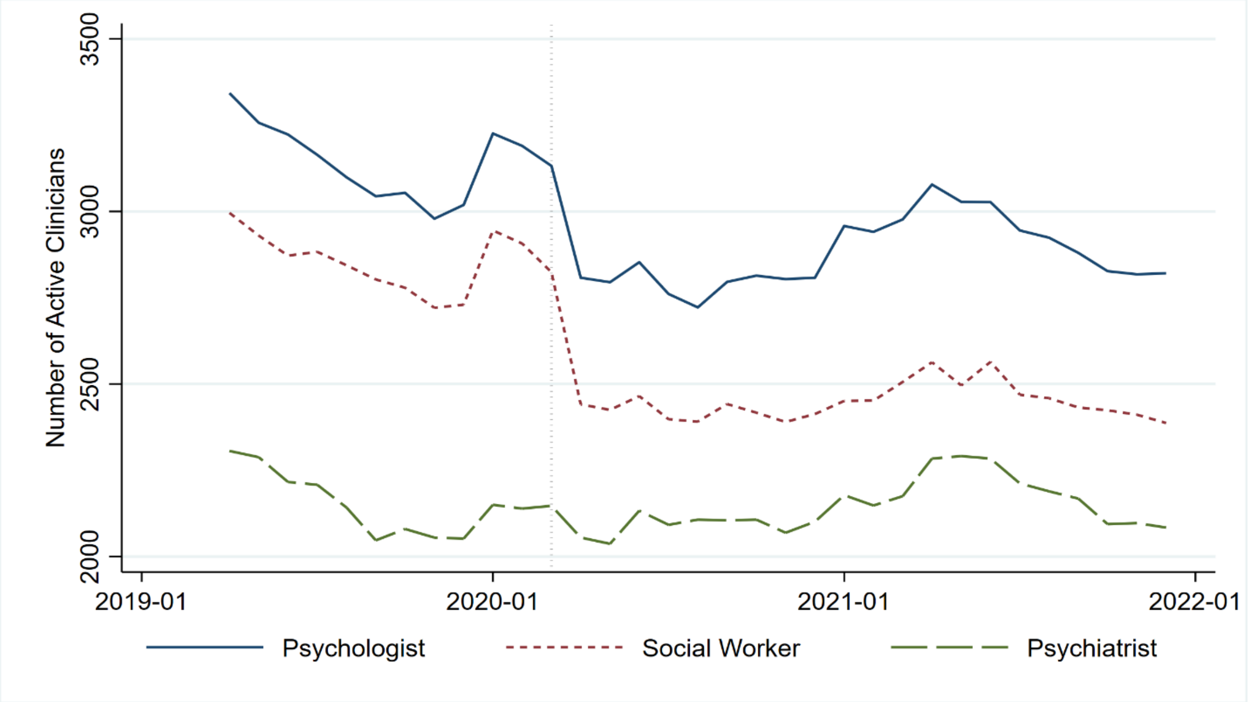

Note: This figure shows the number of active clinicians by type over the study period.

References

1. Carrieri V, Madio L, Principe F. Vaccine hesitancy and (fake) news: Quasi-experimental evidence from Italy. *Health Econ*. 2019;28(11):1377-1382.

2. Van Parys J, Brown ZY. *Broadband Internet Access and Health Outcomes: Patient and Provider Responses in Medicare*. National Bureau of Economic Research; 2023. Accessed June 24, 2024. https://www.nber.org/papers/w31579
